# The Maryland Dental Law

**Published:** 1892-03

**Authors:** 


### Editorial, Etc.

### THE MARYLAND DENTAL LAW.

The January editorial discussed the Maryland Dental Law, and suggested several features deemed desirable to be embraced in the scope of a new bill which, it was understood by all parties in interest, would be presented to the General Assembly of Maryland to supersede the existing dental law passed at the January session, 1884, and amended at the January session, 1886. The following is a copy of the bill introduced during the current month, together with the special correspondence to the Baltimore *Sun* respecting the bill. The bill is entitled an Act to repeal Article thirty-two, of the Code of Public General Laws, entitled "Dentistry," and to re-enact said Article with amendments.

SECTION 1st. Be it enacted by the General Assembly of Maryland, That Article thirty-two, of the Code of Public General Laws, entitled "Dentistry," be and the same hereby is repealed and re-enacted, so as to read as follows:

SEC. 1. It shall be unlawful for any person to practice dentistry in this State, unless he shall have obtained a certificate as herein provided.

SEC. 2. There shall be a Board of Examiners to consist of five reputable practicing dentists, graduates of a recognized reputable dental college or dental department, three of whom shall be from Baltimore city and two from the counties, one shall be from the Eastern and one from the Western Shore counties of the State, whose duty it shall be to carry out the purposes and enforce the provisions of this Article; the members of said board shall be appointed by the Governor, who shall select them from such names as shall be recommended by a mass-meeting of the dentists of the State, called for such purpose by the officers of the Board of Dental Examiners, who shall give a week's notice by postal card or otherwise to all the registered dentists of the State, of the time and place of holding said mass-meeting; provided, that none of said board shall be pecuniarily connected with any dental college or dental department of any college or university; the terms for which the members of said board shall hold their office shall be for four years, and until their successors shall be duly appointed; in case of a vacancy occurring in said board, such vacancy shall be filled by the Governor as provided in this section (section 2.)

SEC. 3. Said board shall choose one of its members President and one Secretary thereof; it shall fix time and place of its meeting or meetings; a majority of said board shall at all times constitute a quorum, but a less number may adjourn from time to time until a quorum is present, and the proceedings thereof shall at all reasonable times be open to public inspection; the board shall also make a report of its proceedings to the Governor annually in the month of January.

SEC. 4. Any person who was engaged in the practice of dentistry in this State prior to the thirty-first day of

March in the year one thousand eight hundred and eighty four, and who shall desire to continue such practice, shall cause his or her name and residence or place of business to be registered with said Board of Examiners, who shall keep a book for the purpose of such registration; the statement of every such person shall be verified under oath before a Notary Public or Justice of the Peace in such manner as may be prescribed by the said Board of Examiners; every person who shall so register with said board as a practitioner of dentistry shall receive a certificate of such registration which shall entitle him or her to continue the practice of the same as such.

SEC. 5. Any person who has graduated and holds a diploma from a university or college authorized to grant diplomas in dental surgery by the laws of some one of the United States, and who is desirous of practicing dentistry in this State, may present the same to said Board of Examiners for approval; such board may, if it shall deem it to be necessary or expedient, examine such person with reference to his or her knowledge and skill in dental surgery, and upon being satisfied as to the qualifications of the applicant and the genuineness of said diploma, shall indorse the same as approved, and, having registered the name and residence of place of business of such person in the aforesaid book to be kept by it for the purpose of registration, shall issue to him or her the certificate hereinbefore provided.

SEC. 6. All certificates issued by said board shall be signed by its officers under seal.

SEC. 7. Transcripts from the book kept as aforesaid by said board for the purpose of registration, certified by the officer who has it in keeping, with the seal of said Board of Examiners, shall be evidence in any court of this State.

SEC. 8. For every certificate issued by said board and for every transcript certified as aforesaid from said book, said board shall charge and receive from the person requiring the same the sum of one dollar, and also shall in like manner charge and receive from every person who shall appear before them for examination as provided in section five of this Article, the sum of five dollars, to be paid to the Secretary of the Board at the time of filing application.

SEC. 9. Any person who shall violate any of the provisions of this Article shall be deemed guilty of a misdemeanor, and upon conviction thereof (in any court having criminal jurisdiction), shall be fined not less than fifty dollars, nor more than three hundred dollars, or be confined not more than six months in the county jail, in the discretion of the court; all fines received under this Act shall be paid into the common school fund of the city or county in which such conviction takes place.

SEC. 10. The officers of said board may grant a certificate to any applicant upon presentation by such applicant of the evidence requisite for obtaining the certificate hereinbefore provided for, shall entitle the person to whom it is issued to practice in this State until the next regular meeting of said board after the granting of said certificate, and no longer, but no such certificate shall be issued by officers after such applicant has been rejected by said board.

SEC. 11. Nothing in this Article shall be so construed as to interfere with the rights and privileges of resident physicians and surgeons.

SEC. 2. And be it enacted, That nothing in this Act shall prevent or be so construed in any way to hinder the prosecution, conviction or punishment of any person who may have offended against any of the provisions of said Article thirty-two, or against any of the provisions of any of the Acts of Assembly of which the same was a codification.

SEC. 3. And be it enacted, That the persons who at the time of the passage of this Act constitute the Board of Examiners provided for by said Article thirty two, shall continue to act for the residue of the term for which they were respectively appointed, and until their successors shall have been duly appointed and qualified.

SEC. 4. And be it enacted, That no member of the Board of Dental Examiners, as created by this Article, shall serve for more than two consecutive terms.

SEC. 5. And be it enacted, That this Act shall take effect from the date of its passage.

---

ANNAPOLIS.—The bill introduced by Mr. Hayes in the Senate, and which is still pending, to repeal and re-enact, with amendments, article 32 of the present Code of Public General Laws, title "Dentistry," serves to direct attention to the present unsatisfactory condition of the State law regulating the practice of dentistry, and cannot be said itself to offer either a satisfactory substitute or remedy. The article of the code, it is admitted, is not expressed in sufficiently precise and accurate terms, but the pending substitute proposes innovations about the policy and even the constitutionality of which there may be grave question. It provides, generally, that no person shall practice dentistry in this State without obtaining a certificate as provided in the act. It then provides that all persons who practiced dentistry in Maryland *prior* to March 31, 1884, may obtain such a certificate by simply registering their names as provided in the act. No examination as to qualifications, &c., is made necessary. It then provides, generally, that all persons having diplomas from dental colleges may obtain a certificate upon satisfying the State board of dental examiners as to their fitness and qualifications to practice dentistry and also as to the genuineness of their diplomas. The examination is optional with the board. There is no saving clause in the bill, as in the bill regulating the practice of medicine, that its provisions shall be construed as *prospective* only. There is the same doubt and uncertainty, apparently, as exists under the present article of the code as to the *status* of dentists who have been engaged in practice *since* the 31st of March, 1884. There is no provision for their obtaining a certificate upon registration merely. Are they required to submit to an examination as to their qualifications, notwithstanding their diplomas and their eight years, it may be, of experience and successful practice? If not, how are they to get their certificates? If yea, what is the reason or justice or constitutional right to discriminate between the dentist who began practice, say on the 25th of March, 1884, and the one who did not begin practice until the 15th of April, 1884, say twenty-one days later? What right has the Legislature to say that the former requires no re-examination, but has only to register his name to obtain his certificate or license to practice, while the latter must submit to be re-examined, at the discretion of the board, as to his diploma and his qualifications? A reasonable law, to apply only to *future* applicants for permission to practice, or one requiring a uniform test from all existing practitioners, without reference to 1884 or any other anterior date, would meet with less adverse criticism and opposition than the present inartifically constructed and badly put together bill.

The following analysis of the bill is given by a well-known dentist: "Senate bill No. 122, recently referred to the committee on judicial proceedings, was doubtless introduced by request, and need not be understood as committing the Senator from Baltimore, who presented it, to all of its provisions. The bill does not emanate from the State board of dental examiners or any incorporated dental body of the city or State, or mass-meeting of dentists. It makes radical changes in the existing dental law, which will be oppressive to *all* the dentists now in practice, requiring them to register again and pay again for a new certificate; it makes a dental diploma a prerequisite for examination, whereas, when a young man has studied law and a board of examiners decided as to his competency he is admitted to the bar; it prevents the appointment by the Governor, even though he elect to appoint men of recognized ability and honor, of any practicing dentists whose names are not recommended by a mass-meeting of the dentists of the State, and at such a mass-meeting four years ago only one dentist from the twenty-three counties of the State attended; it provides that the notice for the mass-meeting shall be given by 'postal card or otherwise,' in the line of Mr. Elkin's famous West Virginia Central by-law, 'at Piedmont or elsewhere,' not through notice in a paper like *The Sun*, but by publication, it may be, (constructive service') in a paper or dental journal that circulates among the dentists of Maryland from the mountains to the ocean about as much as it does in the moon; it provides that the mass-meeting shall be called in like manner every time there is a vacancy; it provides for the 'punishment of any persons who *may have offended*,' thus rendering null and void certificates issued prior to the passage of the act; it prohibits a member of the board to serve longer than two consecutive terms, and then, inconsistently, by another section, continues one member of the board in office for *three* consecutive terms, or ten years; it makes no provision for members absenting themselves from meetings (say from two regular meetings consecutively as is customary in dental laws) as should be in the Maryland dental law, because for lack of a quorum but one meeting has been held since July, 1890, no 'annual report' made to Governor Jackson in 1891 or to Governor Brown in 1892, as the existing dental law requires, and it does not provide, as in all cases should be the rule, that the theoretical examination be submitted in writing and the questions and answers filed, as elsewhere there was 'witnessed the examination of a student at one time by a gentleman who could not answer the questions himself—questions from a quiz-book.' "

---

ANNAPOLIS.—The dentists' bill will have a rather hard time in the Senate. It will probably go to the committee on the sanitary condition of the State, of which Dr. Wootton is chairman, and it must be a mighty correct bill to pass muster before that sterling democrat and anti-monopolist. Dr. Wootton hates monopoly with instinctive aversion, and this bill is monopoly pure and simple. It is in fact opening up a new field for trusts. If a coterie of old dentists can drive all the young men out of the business and close the doors against those who desire to enter they will, of course, have a good thing. It would be a lead which would pan out rich. The bill is heartily denounced by the young dentists of the State and most of the old ones. It is not fair to the profession to charge it with this bill, which is in truth the act of but a small coterie. The bill is different in principle from the medical bill, the latter being much less objectionable, and applies to no one now practicing, while the dental bill reaches its insidious fingers back eight years into the past and gives a dental board formed by a fearful and wonderful method the power to close up the office of any dentist who has gone into the profession within the last eight years. The purpose of the bill, it is said, is to suppress a few "quacks," but at the same time the board can suppress all the young men who have entered the profession since 1884.

### STATE EXAMINING BOARDS.

"A Layman" writes: "It is too late a day to talk about restricting the right of young men to enter the learned professions. It may have been well enough when students acquired their knowledge solely in the offices of private practitioners and went into practice themselves without any other preparation, but now, when our colleges are provided with learned and experienced faculties and all the facilities which modern science commands, it is both superfluous and absurd to set up State boards of examination to review their work. Under the best conditions such a review could not be fairly done. Students pass two or three years under the immediate supervision of their preceptors, who know them all over and have measured their qualifications with as much accuracy as it is possible for them to be measured. A few incompetents may slip through, but the very same incompetents can even more easily pass a board of examiners who don't know anything about them and who are very likely as in competent as they are. My observation of such boards in several States is that they are more frequently animated by a desire to 'thin out' the profession than to improve its personel or protect the public. In fact, the boards themselves are very rarely composed of competent men, no matter whether chosen by associations or by political discretion. The best class of practitioners will have nothing to do with them."

"Dentist" writes to the *Sun* as follows: "It is but fair to the dentists of the city and State to say that the bill introduced into the Senate last week repealing the existing dental law had not been submitted to or approved by the incorporated bodies of the city and State, the Association of Dental Surgeons or the State Dental Society."

